# Autonomy Benefits and Risks of Assistive Technologies for Persons With Intellectual and Developmental Disabilities

**DOI:** 10.3389/fpubh.2018.00296

**Published:** 2018-11-02

**Authors:** Fiachra O'Brolcháin

**Affiliations:** School of Theology, Philosophy and Music, Dublin City University, Dublin, Ireland

**Keywords:** assistive technology, intellectual disability disorders, autonomy, ethics, philosophy, medical ethics

## Abstract

This paper explores the ways in which assistive technologies (ATs) can both promote and undermine the autonomy of Persons with Intellectual and Developmental Disabilities (PIDD). Following an initial discussion of ATs for PIDD, I examine the specific issues of autonomy for PIDD. I outline the ways in which ATs can boost autonomy, of PIDD, focusing on knowledge, authenticity, and liberty. Following that I suggest that ATs are not necessarily beneficial in terms of autonomy and examine ways that they might be used to undermine the autonomy of PIDD, specifically the categories of knowledge, authenticity, and liberty. I conclude by suggesting that the development of ATs requires ethical oversight.

## Introduction

Assistive technologies (ATs) offer much potential to improve the lives of persons with intellectual disabilities. These technologies,^1^ which can range from the simple (handheld digital magnifiers) to the extremely sophisticated (e.g., brain-computer interfaces), are being designed to help persons with disabilities of all sorts to function more easily in the world. The range of persons with disabilities is extensive and the number is increasing. It can include persons with physical disabilities, intellectual disabilities, and cognitive impairments.

This paper focuses on the subset of ATs being designed for persons with intellectual and developmental disabilities (PIDD). Intellectual disabilities are a multifaceted phenomenon with many variations, and there is a wide range of ATs being developed. While ATs are designed with many goals in mind, many are ultimately intended to increase the user's autonomy.

Within Western philosophical thought, respect for individual autonomy is a core principle. Within Western medical ethics, there is a broad consensus that respecting patient autonomy is required for good practice. That autonomy is a goal of those developing ATs should not surprise us then, as it is becoming a cornerstone of ethical thinking in medical contexts. Nonetheless, it is worth examining what is meant by autonomy in the context of ATs. Few people will argue against the goal of promoting the autonomy of PIDD, making this goal a useful claim for tech developers wishing to promote their products. This paper intends to examine the ways in which ATs can both promote and undermine the autonomy of PIDD. This involves providing a more detailed account of what is meant by autonomy and exploring the ways ATs will interact with the conditions essential to it.

This discussion is emblematic of a larger discussion regarding the development of novel technologies. New technological developments, such as Big Data, nudging, internet-of-things, are better able to attract our attention (which is considered to be a finite resource). The more privacy we surrender, the more information those with access to technology have about us, and the easier it is for them to nudge us to behave in certain ways. As well as raising deep philosophical questions about the nature of human freedom (which, unfortunately, is not addressed here due to lack of space), this raises political questions about the control of and access to novel technologies. The positive outcomes of assistive technologies are a major justificatory reason for developing such technologies. However, as we see in this paper, there also exist hidden dangers. As such, the discussion of the impacts of ATs for PIDD has implications for the wider population. While the technologies might first be used to help PIDD, they are likely to become more widely available; and while the vulnerabilities of PIDD are more obvious, similar vulnerabilities exist in us all. Technological companies thrive by attracting people's attention, which enable them to more easily sell to those people. PIDD are in a number of ways more attentionally vulnerable, but the issue is not theirs alone. This is an ethical issue for everyone.

The structure of the paper is as follows. First, a brief outline of the goals of autonomy is provided, followed by an introduction to PIDD and the types of ATs being developed for PIDD. Following this, the issues of autonomy in relation to PIDD is introduced. The next section outlines the ways in which ATs might promote or boost the autonomy of PIDD. The following section suggests some ways in which the ATs might undermine the autonomy of PIDD. There is then a brief discussion and conclusion.

As a philosophical paper, this paper relies on conditionals. ATs *may* bring benefits and *may* bring harms. The future is not set and the overall result of ATs is and will be dependent on numerous factors, ranging from technological developments to policy makers, regulators, and users. This paper aims to elucidate the philosophical concept of autonomy and explore the possible real impacts of ATs on autonomy, so that developers, policy makers, regulators, and users will have a better understanding of the impact of ATs.

## The goal of autonomy

ATs are frequently advocated as aids to increase the autonomy of people with intellectual disabilities (PIDD). Increasing or improving the autonomy of PIDD is a goal of the United Nations Convention on the Rights of Persons with Disabilities [(1): Preamble (n)]. The same convention obliges signatories to “undertake or promote research and development” of “assistive technologies” [(1), 4, g, h]. The Convention does not make a link between the use of assistive technologies and autonomy, but this connection is prevalent in the literature about the use of ATs and PIDD (2, 3). For instance, a person's autonomy is one of the factors measured in determining an individual's predisposition to use AT (4). A good example of this is the University of Victoria's CanAssist program which focuses on developing and deploying assistive technologies for students with disabilities and aims to remove barriers to inclusion and to create tools that will provide persons with disabilities with greater autonomy and independence [(5), p. 57].

## Persons with intellectual and developmental disabilities and assistive technologies

Intellectual and developmental disabilities cover a wide range of conditions. The US National Institute of Health defines intellectual disability as starting before a person turns 18 and consisting of “intellectual functioning or intelligence, which includes the ability to learn, reason, problem solve, and other skills”; and “problems with adaptive behavior, which includes everyday social and life skills” (6), while developmental disabilities might encompass a broader range of disabilities, both physical and intellectual. Intellectual disability can range from mild to severe or radical. While mild cognitive disability would include learning difficulties or attentional disorders, severe cognitive disability might “limit or preclude the development of … the consciousness of oneself as a temporally-extended being; practical rationality—the capacity to govern one's actions by reasoning about how to act; and the capacity to make and respond to moral demands” (7). These latter attributes are sometimes considered essential if a person is to be considered to have full moral status (7), and thus are of much theoretical, practical, and emotional importance. Thus, cognitive disability is best viewed as being a spectrum. Similarly, being autonomous is, as will be discussed in the next section, also predicated on certain attributes, and similarly autonomy can be viewed as being on a spectrum.

Technological aids are therefore of immense importance as they may bring people up to a certain threshold, whereby they will be better able to communicate, interact, or be part of their society. There is, understandably, much excitement about this potential. Assistive technologies similarly cover a wide-range of devices and equipment that facilitates teaching new skills, augments existing skills, or otherwise reduces the impact of disability on daily functioning [(8), p. 157]. It may be extremely high-tech (making use of virtual reality, robotics, or brain-computer interfaces) or low-tech (pencil grips, slant boards, pictures for communication). In between are technologies such as microswitches, which facilitate persons with certain disabilities to communicate via very simple responses (e.g., small movements of their hands/fingers, lips, or eyelids). This allows PIDD to “(a) access a computer system and choose and activate different program options or (b) activate simple environmental stimulation (i.e., depending on their levels of intellectual functioning and engagement interests)” (9).

Different ATs are likely to be used for people with mild, moderate, and severe disabilities. For people with mild disabilities, reminders, and guides would be applicable; it is similar for people with moderate disabilities, who might also make use of ATs that help with communication, emotional skills, or adaptive skills in daily living. Communication devices might also be useful for people with severe ID. Other ATs for people with moderate or severe ID might be used to control impulses or discern their preferences. As technologies advance, it should be possible to develop technologies that will be personalized and more precise. These may be better able to help those with severe ID.

## Autonomy of people with intellectual disabilities

Personal autonomy, according to Beauchamp and Childress [(10), p. 101]. “encompasses self-rule that is free from both controlling interference by others and limitations that prevent meaningful choice, such as inadequate understanding”. We can think of autonomy in the sense of being free from the control of other people or groups, e.g., autonomy in the political and social sense, or we can think of autonomy in a more personal sense intimately connected with notions of authenticity. Autonomy in the political and social sense, i.e., autonomy as it relates to a person's choices, is not the primary concern of this paper, although there are connections between political autonomy and autonomy in the sense of it being a feature of a person's self. Suffice it to say that if we assume the basic principles of liberalism, our aim will be to remove as many limitations on choices as possible. ATs can benefit this type of autonomy in relation to PIDD, as will be discussed below. ATs can certainly improve some aspects of personal autonomy, i.e., autonomy as it relates to authenticity and self-rule, but in other ways might undermine it. ATs will alter the conditions in which values, choices, and plans are made and the media through which they are facilitated and communicated. The question that is worth asking concerns the impact ATs will have on the autonomy of people, including PIDD.

This is not to say that a PIDD or the choices, values, and plans of PIDD should be afforded any less respect or ethical value. However, there will be reason for concern if those choices, values, and plans are not “theirs.” The normative judgements about the autonomy of PIDD using ATs may be especially relevant in relation to PIDD for a number of reasons as follows:

1. There is a push, as we have seen, for ATs to be developed for PIDD (carers and others want to see PIDD live more autonomously; grand claims are being made regarding the promise of ATs).
2. PIDD may be unlikely to be able to evaluate as fully the risks and benefits of ATs for their autonomy, depending on the level, and severity of their ID.
3. carers and others who are emotionally involved with PIDD may be blinded by the promise of the ATs.
4. If PIDD are considered to be closer to full autonomy, they ought to be autonomous in a political sense as well [c.f. (11)]. That they vote according to their own conscience is of the utmost importance.

None of this is to suggest that ATs should not be developed for PIDD nor that PIDD should not be permitted to vote or have any other civil and political rights. While Feinberg (12) has identified four different variations of autonomy in moral and political philosophy, central to all of them “is a conception of the person able to act, reflect, and choose on the basis of factors that are somehow her own (authentic in some sense)” (13). Certain types of ATs pose a threat to this type of autonomy. This will be discussed in more detail below.

Given the different understandings surrounding the concept of autonomy, it will be useful to start with a working definition. We can then say that at minimum, autonomy consists of self-rule, of deciding for oneself what one would like to be able to do. As such three components stand out: (1) knowledge, (2), authenticity, and (3) liberty. Knowledge will be required in order for a person to make decisions about what he or she values, as well as being essential if one is to determine what one wants to do in pursuit of their values. Authenticity is then required if the person is to be the initiator of his or her own actions—if a person is merely following someone else's plan or is brainwashed, then he or she is not fully autonomous. Here, liberty means political autonomy or having rights over themselves.

## Assistive technologies and the promotion of autonomy

In relation to ATs, Leslie Francis notes that people with physical disabilities use assistive devices, but his or her actions involving these devices are still considered to be his or her own; she also correctly notes that people without intellectual disabilities also use assistive devices. So, the fact that someone might use an assistive device does not necessarily mean that they have diminished autonomy. Francis contends that

“…the significance of prostheses and other forms of assistance is normative. The judgement that a value, or a choice, or a plan of action is not sufficiently or appropriately a matter of my individual psychological processing to be regarded as ‘mine' is a normative conclusion about how that value, or choice, of plan of action is to be regarded, whether it is to be respected, how I am to be treated in light of it” [(14), p. 208].

Clearly, using assistive technologies does not mean that a value, choice, or a plan is insufficiently a matter of a person's individual psychological processing to be regarded as theirs. Francis is arguing against a perception that PIDD lack autonomy and is pointing out that a person's use of ATs should not be held against him or her. Nonetheless questions over whether someone's values, choices, or plans of action are a matter of their individual psychological processing is of great ethical importance including in terms of assistive technologies, and including (more specifically) assistive technologies for PIDD.

Let us first examine the many ways in which ATs could promote autonomy. Take for example the number of deficits faced by at least some PIDD that relate to autonomy listed by Francis: difficulty with abstract reasoning, difficulty with impulse control, difficulty in planning ahead and in pursuing developed plans, problems in social adaptation that might manifest as gullibility, naiveté, and increased and potentially problematic dependency on others [(14), p. 204]. Technological aids could help PIDD overcome or manage a number of these deficits, thus promoting autonomy.

Francis points out that, “that what it is to have autonomy in some relevant senses is a complex matter and that judgements about the autonomy of people with intellectual disabilities must be complex as well” [(14), p. 200–201]. She provides a more extensive list of attributes of autonomy: “being able to value, being able to reason, being able to resist impulses, being able to imagine an ordered life, being able to order one's life being able to put one's plans into practice, being able to participate in moral deliberation of an idealized kind, and being politically free” [(14), p. 202]^2^. In what follows, the ways in which ATs may be able to promote three subsets of autonomy—knowledge, authenticity, and liberty—are outlined.

### Knowledge

A number of PIDD have difficulty retaining information, so technological aids that supply knowledge in an easily digestible form will be of immense use. Maps and guides are obvious examples, but technology that informs PIDD of what various signs and signals do will also be important, e.g., a device that reminds PIDD about the meaning of road signals might help PIDD walk about a city by themselves. ATs might be used to provide reminders of what their long-term goals are, thus nudging them to align their current desires with previous plans or preferences (possibly made in conjunction with family members or carers). As such, ATs could help with impulse control. ATs might also help with abstract reasoning, by making abstract concepts comprehensible to PIDD.

### Authenticity

Many people with severe ID struggle to “formulate, articulate, or communicate complex ideas” [(15), p. 313]; some are arguably incapable of formulating, articulating, and communicating complex ideas. As such, it is extremely difficult to determine what, if any, their preferences are. In some cases, it will be difficult to determine the authentic desires of people with severe cognitive impairments. For example, a person with severe apraxia might struggle to communicate a sentence, perhaps taking an hour to complete the sentence. While ATs could help, they could also easily distort what the person is likely to say. ATs that aid their communication or that are able to interpret their preferences (e.g., using eye-tracking or emotion recognition software) will help in allowing these people to pursue their authentic goals. If they are able to communicate, then the goals they are given or that they choose are likely to reflect their interests and preferences.

This presupposes that they can be said to have preferences from which interests can be generated. ATs might also be useful in helping PIDD (particularly those with moderate or mild impairments) determine their preferences and order their lives. Assistive devices can be used to make up for deficits in planning ahead and pursuing goals.

Some PIDD have poor impulse control—ATs may be able to promote being one's own person by helping people choose courses of action that should help them achieve their goals in life rather than being led by their intuitive and emotional responses to things. ATs could be used to nudge PIDD toward certain goals (ideally worked out in conjunction with the user). Nudging works by appealing to people's automatic affective systems (i.e., the systems governed by emotional and intuitive reactions to things) in order to promote goals that people would (ideally) choose if they had time to reflect on their longer-term, strategic goals (16). This is relatively easy to illustrate—suppose a person's long-term goal is to eat more healthily in order to live longer, but his or her affective self responds to a cake at the counter by buying the cake. A nudge in such a scenario would arrange the presentation of food so that the cake is not as tempting; an AT might remind the person of their longer-term goal of not eating the cake, thus helping the person achieve this goal; or might suggest lunches; or might calculate the calories of the items that the person buys. Interesting research has been done, for instance, on the links between harmful use of alcohol and tobacco and the density of outlets supplying such products (17, 18).

In terms of PIDD, this sort of nudging is trickier as in some cases of intellectual disability the capacity to form long-term strategic goals might not exist. However, Francis and Silvers have suggested “the possibility of constructing individualized conceptions of their good by, with, and for people with lifelong intellectual disabilities” (14). They argue further “people with intellectual disabilities can participate in practices that are centered on their own ideas of the good, even though they cannot formulate, articulate, or communicate complex ideas” [(15), p. 313]. There is an earlier argument advanced by Francis together with Silvers that “To the extent that people with intellectual disabilities have recognizable preferences, and interests that can be generated from these preferences, other people can collaborate with them to construct individualized, subjective conceptions of the good” [(15), p. 325].

### Liberty

Regarding the liberty condition of autonomy, ATs should reduce PIDD's dependency on others and render them slightly less naïve and gullible in practice (by reminding them how to interact with others, for instance). Moreover, insofar as ATs promoting the autonomy of PIDD in the sense of character or personality, i.e., giving PIDD more knowledge and facilitating their independent pursuit of their own conceptions of the good, there will be a stronger argument for them to enlarge the scope of rights over themselves. For instance, Nussbaum points out that regarding political entitlements, “People with limited ability to read, people who easily become confused or fearful in a new setting, may be excluded from voting and jury service de facto, even though sensitive thought about how to include them could prove just as successful in these settings as it has in education” [(11), p. 344]. By helping PIDD communicate, understand information, and deal with new scenarios and new people, the range of choices available in social and political spheres is increased and thus this aspect of his or her autonomy is increased.

For those who argue for relational autonomy, having normative authority over one's central values and commitments is seen as being of utmost importance, i.e., being the validating source of these values, if not necessarily the origin [(19), p. 375]. In this case, then, so long as PIDD validated the values being promoted by the ATs, they would be seen as autonomous in the relational sense. ATs, by helping PIDD interact with the rest of society more easily, will facilitate their social recognition, and thus promote self-respect and dignity.

## Assistive technologies undermining the autonomy of users

ATs might undermine the authenticity of PIDD. Let us focus on the ways in which ATs might impact the knowledge, authenticity, and liberty conditions of autonomy. While “compulsion, temporary or protracted mental disability, and addiction are most often mentioned as examples” of ways in which autonomy can be undermined, “brainwashing and manipulation of the psychic economy of the agent” are also threats as they “undercut the person's ability to reflectively accept her first-order motives and/or the way that she has come to develop them” [(19), p. 375]. This is not to say that all interference or suggestion undermines autonomy. Persons with IDD can be naïve and not well-informed regarding their use of technologies, making them especially vulnerable. For instance, a study on the use of IT by PIDD found that “privacy breaches were revealed to be a major risk for persons with IDD, who did not seem to consistently understand that they should protect their personal information and *how* it could be used by third parties” (20).

### Knowledge

Electronic ATs (i.e., apps on mobile devices) might distort or misrepresent information being made available for users. Often the information being made available for PIDD will necessarily be simplified so as to make it comprehensible. Depending on the degree of simplification, this could result in misrepresentations of knowledge. The choices made in terms of what information to omit or what to emphasize could be seen as distortion. Designers of ATs will also need to ensure they minimize their own biases.

### Authenticity

The addition of a layer of technology to the relationship between PIDD and the world around them might complicate matters as much as simplify them. We mentioned that some apps might help PIDD communicate their inner psychological states to others. There are potential drawbacks to this ostensible benefit—the risk that the user's inner psychological state will be misrepresented. It may be distorted or manipulated in translation. In cases of people with profound cognitive impairments, it might be extremely difficult to determine if the AT is in error or if their preferences have changed.

Secondly, if an app—for instance—offers the user a range of options from which to choose, the choice architecture [c.f. (21)] will nudge them in one way or another. The degree of nudging, which is unavoidable, risks undermining their autonomy.

Of course, this is not only the case for PIDD. Recent psychological research suggests that “the palatability of certain kinds of mental stimulation seems to be hard-wired, just as our taste for sugar, fat, and salt is” [(22), p. 16]. Companies in possession of huge amounts of data about a person that develops apps for that person will be easily able to provide us with stimulation that we crave, thereby undermining capacities for self-regulation (which is said to be finite) as well making claims on our attention. Novel technologies are being designed to target the “attentional resources” of people, resources that are considered finite [(22), p. 11]. Moreover, the ability to allocate attention is linked with self-regulation, meaning that “To the extent that the power of concentration is widely attenuated, so too is the power of self-regulation” [(22), p. 16]. The less attentional resources we are able to muster at any time, the more pliable we become and the more vulnerable we are to manipulation. Novel technologies, by gathering data about us, are better able to determine which nudges and techniques will grab our attention. The less we are able to focus on ourselves, the more easily manipulated our decisions and choices will be—the less “authentic.” This impacts on autonomy, including the autonomy of PIDD. So, while ATs might provide more knowledge and better ability to communicate, it will be essential to ensure that they do not reduce PIDD's “attentional resources.”

ATs might be used either to help determine what a PIDD's preference are, or to create those preferences. If some third party creates the preferences, it becomes difficult to claim that the PIDD is autonomous.

### Liberty

If a PIDD has no (or severely reduced) personal autonomy in the sense of them initiating their own actions, developing, and following their own plans, or choosing their own values, then they cannot be said to have very much autonomy. As such, there would be severe problems with political autonomy. If they were not authentically choosing how to vote, for instance, but were instead voting according to preferences suggested to them by an AT, or making jury-deliberations, there would be a risk to the legitimacy of those processed. A major risk of ATs then is that they might be used to manipulate PIDD for legal or political ends. If this were to happen it would set back the cause of obtaining equal civil and political rights for PIDD.

Less individualistic accounts of autonomy such as relational accounts (23) of autonomy do not necessarily help in this case either. Relational accounts of autonomy focus on recognition of the person's autonomy, and on the importance of a person validating their own values. However, this simply shifts the problem—ATs could nudge the person toward validating the values the AT suggests. The user might be seen not as the origin of these values but as validating them. If they have been nudged toward validating them, then the problem remains.

## Discussion

Our notions of autonomy emphasize the importance of individuals making choices according to their own individual preferences. Indeed, a core tenet of political liberalism is that people should be allowed to pursue their own subjective account of the good. Recent developments in big data technologies, alongside developments in “nudging” techniques, mean that our preferences themselves are increasingly subject to social engineering.

In developing ATs for PIDD, we must be extremely careful to ensure that novel technologies do not in fact create preferences based on the interests of technology designers, engineers, or carers. Francis and Silvers argue that a conception of the good “can be carried on, and indeed often is carried on, in dependency” [(15), p. 313] (e.g., with others) and seem to envisage carers or other trusted people helping PIDD develop conceptions of the good. They note that “For a conception of the good to be individually scripted, it must be tailored to the individual in a way that reflects the individual's subjective experiences and personal characteristics” [(15), p. 322]. ATs can help carers understand and interpret the preferences of PIDD and help PD communicate those preferences [c.f. (24, 25)]. As such, they are useful, but they also risk misrepresenting an individual's subjective experiences, or constructing preferences (by framing responses to certain things or directing their attention in specific ways) that are in fact in the interests of the developer. In scenarios where the PIDD has a profound impairment, they will struggle to correct it; or ATs will be able impact on the PIDD's subjective experiences. Although ATs will bring benefits then, they will also present PIDD and carers with novel problems and threats.

The issue of ATs undermining the autonomy of PIDD will be most pronounced in cases of profound cognitive impairment, as it is in these cases that it is difficult to determine the preferences of PIDD and their degree of autonomy. ATs are likely to promote the autonomy of PIDD at the mild or moderate end of the spectrum. They are likely to facilitate greater independence by serving as memory aids, guides, and planners. They will certainly promote autonomy in this sense. They may help people develop and pursue life goals. In terms of people with moderate or severe ID, they can promote autonomy in the sense of providing means to communicate and to control impulses.

In all cases there remains a risk that they will also make users biased toward or against particular goals. The degree to which nudging undermines autonomy is pressing then.

ATs insert an extra-layer into decision making for people with severe ID. While Silvers and Francis have argued that it might be possible to determine someone's subjective good, this relies on others to do so. ATs may help in this quest, but they also complicate the matter, as they can be used to interpret the preferences of the user. Given the epistemological uncertainty regarding the preferences of a person with severe ID, a reliance on ATs will still require the involvement of guardians or carers.

This suggests the need for strict controls on the development of ATs, at least for those involved in certain stages of AT development, just as people working in care are strictly vetted. At the very least, increased ethical oversight is required. The aim of promoting the autonomy of PIDD is commendable and ATs are, at first glance, immensely appealing. However, if we are to actually promote autonomy, we need to analyze carefully what is meant by autonomy, and ensure that those conditions are not undermined by the very technologies designed to increase autonomy.

## Conclusion

This paper discusses the ways in which ATs can both promote and undermine the autonomy of PIDD. Following an initial discussion of ATs for PIDD, issues of autonomy for PIDD were addressed. The ways in which ATs can boost autonomy of PIDD, focusing on knowledge, authenticity, and liberty, were outlined. It was then suggested that ATs are not necessarily beneficial in terms of autonomy and that they might be used to undermine the autonomy of PIDD, specifically the categories of knowledge, authenticity, and liberty. It can be concluded by suggesting that the development of ATs requires ethical oversight.

However, this discussion touches on broader issues that pose ethical issues for every facet of society, not just for PIDD and those who care for them. Threats to our attentional resources are becoming more apparent and risk making people more pliable and threatening their individual autonomy. Given the centrality of individual autonomy to democracy, this is an issue that all democratic governments should be addressing. A blinkered focus on the positive potential of new technologies, for instance the potential of ATs to promote the autonomy of PIDD, risks blinding us to their dangers. The financial incentives associated with the creation of novel technologies mean they are inevitable, but if we are to avoid the risks and maximize the benefits, more ethical oversight and (probably) regulation will be needed.

## Author contributions

The author confirms being the sole contributor of this work and has approved it for publication.

### Conflict of interest statement

The author declares that the research was conducted in the absence of any commercial or financial relationships that could be construed as a potential conflict of interest.

## Footnotes

^1^If we adopt a very broad definition of ATs, canes and crutches, spectacles, and wheelchairs, and other venerable and familiar technologies count as ATs.

^2^She also outlines a number of deficits faced by at least some PIDD that relate to autonomy, such as difficulty with abstract reasoning, difficulty with impulse control, difficulty in planning ahead and in pursuing developed plans, problems in social adaptation that might be manifest as gullibility, naiveté, and increased and potentially problematic dependency on others [(14): p. 204].
